# Diagnosis of abdominal tuberculosis by mini-laparoscopy

**DOI:** 10.1007/s15010-022-01800-3

**Published:** 2022-03-18

**Authors:** Thomas Theo Brehm, Stefan Schmiedel, Ansgar W. Lohse

**Affiliations:** 1 I. Department of Internal Medicine, University Medical Center Hamburg-Eppendorf, Martinistraße 52, 20246 Hamburg, Germany; 2 German Center for Infection Research (DZIF), Partner Site Hamburg-Lübeck-Borstel-Riems, Hamburg, Germany

**Fig. 1:**
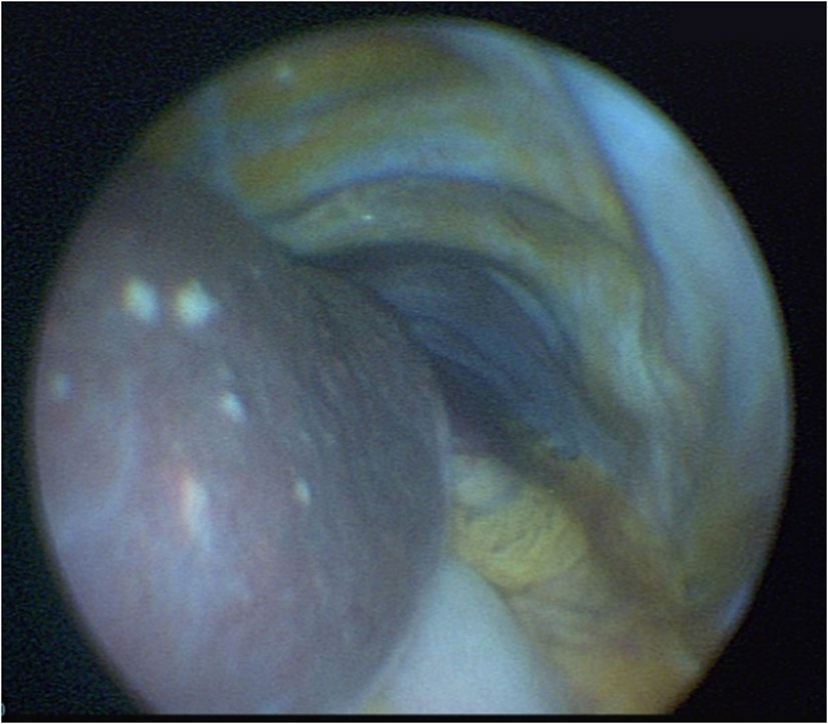
Minilaparoscopy showing multiple whitish nodules scattered over the liver

An 86-year-old woman presented with a 4-week history of fever and unexplained weight loss. Her medical history was notable for psoriasis vulgaris, and treatment with the TNF inhibitor adalimumab was initiated 3 months earlier. Laboratory examination revealed elevated AST (82 U/l), ALT (59 U/l), C-reactive protein (164 mg/l), and erythrocyte sedimentation rate (77 mm). A mini-laparoscopy was performed as described previously using a 2.75 mm trocar, a 2.3-mm Veress needle, and a 1.9-mm laparoscope [1]. It revealed multiple, small, whitish nodules scattered over the liver and spleen (Fig. 1). Hepatic biopsies showed acid-fast bacilli by direct microscopy, tested positive for *Mycobacterium tuberculosis* by PCR and mycobacterial culture later revealed a fully susceptible *M. tuberculosis* strain. The patient was placed on a standard regimen with rifampicin, isoniazid, ethambutol, and pyrazinamide and had a good clinical response to treatment. This case demonstrates that mini-laparoscopy is a valuable tool in the diagnostic workup of patients with suspected abdominal tuberculosis, which is notoriously challenging due to nonspecific clinical, laboratory and radiological features [2]. Laparoscopic techniques offer the opportunity of targeted biopsies for histopathologic and microbiologic analyses under visual control and have a high diagnostic yield in patients with abdominal tuberculosis [3]. Compared to conventional laparoscopy, mini-laparoscopy is a less invasive technique, which requires smaller insertions to the abdominal wall due to ultra-fine instrumentation and can be safely conducted outside of the operation room with the patient under conscious sedation.
